# Two Cases of Resected Bronchiolar Adenoma/Ciliated Muconodular Papillary Tumor Requiring Differentiation from Peripheral Lung Cancer

**DOI:** 10.70352/scrj.cr.26-0052

**Published:** 2026-04-22

**Authors:** Yu Sugimoto, Takeshi Hanagiri, Syuhei Ashikari, Takashi Iwanami, Jongkun Park

**Affiliations:** 1 Department of Thoracic Surgery, Kitakyushu General Hospital, Kitakyushu, Fukuoka, Japan; 2 Department of Diagnostic Pathology, Kitakyushu General Hospital, Kitakyushu, Fukuoka, Japan

**Keywords:** bronchiolar adenoma/ciliated muconodular papillary tumor, video-assisted thoracoscopic surgery, frozen section, lung adenocarcinoma

## Abstract

**INTRODUCTION:**

Bronchiolar adenoma/ciliated muconodular papillary tumors (BA/CMPTs) are rare benign tumors. Because their radiological appearance often resembles that of small peripheral lung cancers, preoperative differentiation remains challenging. Furthermore, BA/CMPTs may be misdiagnosed as adenocarcinomas during intraoperative frozen-section consultations. Here, we report two cases of resected BA/CMPTs with distinct radiological morphologies and discuss their clinicopathological characteristics compared with those of previously reported cases.

**CASE PRESENTATION:**

The first case involved a 69-year-old man who presented with an 8-mm solid nodule in the right lower lobe. Primary lung cancer (cT1aN0M0, cStage IA1) was suspected, and video-assisted thoracoscopic wedge resection of the right lower lobe was performed for diagnostic and therapeutic purposes. Intraoperative frozen section evaluation suggested adenocarcinoma; however, no additional resection was performed in accordance with a planned limited approach considering the comorbidities, including chronic kidney disease. Permanent sections revealed a bland bilayered epithelial proliferation with focal ciliated epithelium. Immunohistochemical analysis revealed a continuous basal cell layer positive for CK5/6 and p40, leading to the final diagnosis of BA/CMPT. The second case involved a 77-year-old man who presented with a 9-mm pure ground-glass nodule (GGN) in the right lower lobe. Early lung adenocarcinoma (cTisN0M0, cStage 0) was suspected. Following preoperative CT-guided marking, video-assisted thoracoscopic wedge resection was performed. Permanent sections showed no significant cytological atypia, focal ciliated epithelium, or a continuous basal cell layer highlighted by CK5/6 and p40, confirming BA/CMPT. After complete resection, both patients were followed up without adjuvant therapy.

**CONCLUSIONS:**

BA/CMPTs can present with diverse radiological appearances, ranging from small solid nodules to pure GGNs, and typically show slow interval growth, making their differentiation from small peripheral lung adenocarcinoma challenging. Moreover, the evaluation of cilia and the basal cell layer is often limited in frozen sections, which may lead to misdiagnosis as adenocarcinoma. Therefore, awareness and shared recognition of this entity is essential to avoid overtreatment such as lobectomy and systematic lymph node dissection. Further accumulation of cases is warranted to refine the diagnostic criteria and optimize the management strategies for BA/CMPTs.

## Abbreviations

BA/CMPTs
: bronchiolar adenoma/ciliated muconodular papillary tumors
CEA
: carcinoembryonic antigen
CYFRA
: cytokeratin 19 fragment
GGN
: ground-glass nodule
NSE
: neuron-specific enolase

## INTRODUCTION

BA/CMPTs are rare benign tumors that were newly described in the 5th edition of the WHO Classification of Thoracic Tumours (2021). Their radiological appearance often resembles that of small peripheral lung cancers, making preoperative differentiation a clinical challenge. In addition, intraoperative frozen section consultation may lead to misdiagnosis because of the inherent limitations of frozen section evaluation. Here, we report two cases of resected BA/CMPTs with distinct radiological morphologies and compare their clinicopathological characteristics with those described in previous reports.

## CASE PRESENTATION

### Case 1

**Fig. 1:**
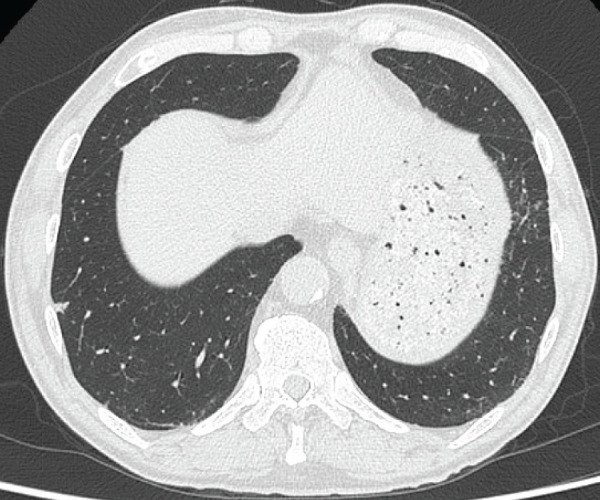
Chest CT showing an 8-mm solid nodule in the peripheral segment 8 (S8) of the right lower lobe (Case 1).

**Fig. 2:**
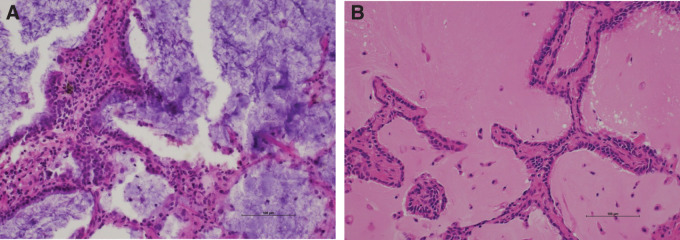
Intraoperative frozen-section findings in Case 1 (original magnification, ×200). (**A**) H&E-stained frozen section; ciliated cells are not clearly identifiable. (**B**) H&E staining of the same tissue after formalin fixation (×200), in which ciliated epithelium is more readily appreciable. H&E, hematoxylin and eosin

**Fig. 3:**
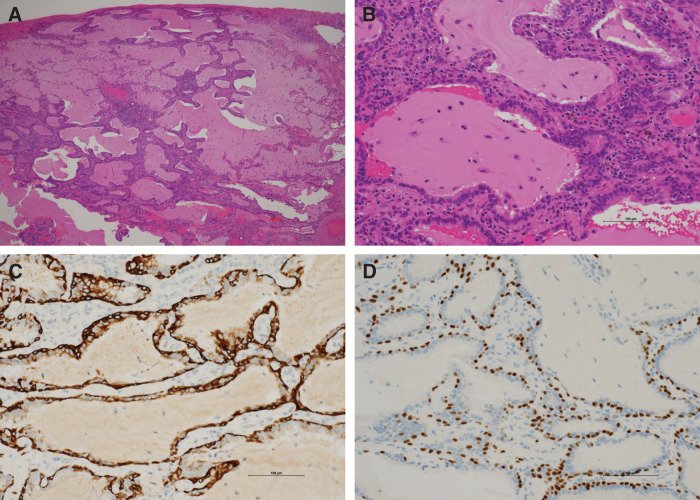
Histopathological and immunohistochemical findings in Case 1. (**A**) Low-power H&E staining (original magnification, ×40) showing bland epithelial proliferation along the alveolar walls. (**B**) High-power H&E staining (×200) demonstrating bland bilayered epithelium with focal ciliated epithelium. (**C**) CK5/6 immunostaining (×200) highlighting a continuous basal cell layer. (**D**) p40 immunostaining (×200) confirming the continuous basal cell layer. H&E, hematoxylin and eosin

A 69-year-old man was found to have an 8-mm solid nodule in peripheral segment 8 (S8) of the right lower lobe (**Fig. 1**), which demonstrated slow growth over a 3-year period. Serum tumor markers (CEA, CYFRA, and NSE) were within normal ranges. PET-CT showed no significant uptake (maximum standardized uptake value [SUVmax] = 0.92). Although benign lesions such as hamartomas or inflammatory changes were considered, primary lung cancer (cT1aN0M0, cStage IA1) could not be excluded. Therefore, video-assisted thoracoscopic wedge resection of the right lower lobe was performed for diagnostic and therapeutic purposes. Intraoperative frozen-section evaluation did not clearly demonstrate ciliated cells (**Fig. 2A**), leading to an interpretation of adenocarcinoma. However, considering comorbidities, including chronic kidney disease, no additional resection was performed in accordance with the planned, limited approach. In permanent sections, bland epithelial cells proliferated along the alveolar walls with a focal ciliated epithelium (**Fig. 3A**, **3B**). Immunohistochemistry revealed a bilayered epithelium with a continuous basal cell layer highlighted by CK5/6 and p40 (**Fig. 3C** and **3D**), leading to the final diagnosis of BA/CMPT. The postoperative course was uneventful, and the patient was discharged on POD 7. He was followed up without treatment and remained recurrence-free for 1 year after the surgery.

### Case 2

**Fig. 4:**
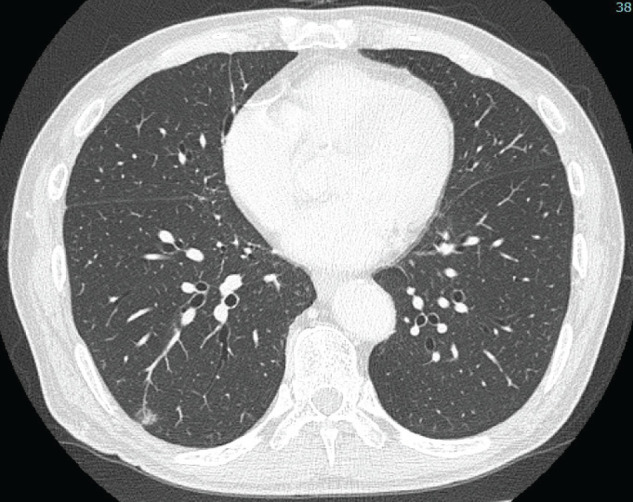
Chest CT showing a 9-mm pure GGN in segments 8/9 (S8/9) of the right lower lobe (Case 2). GGN, ground-glass nodule

**Fig. 5:**
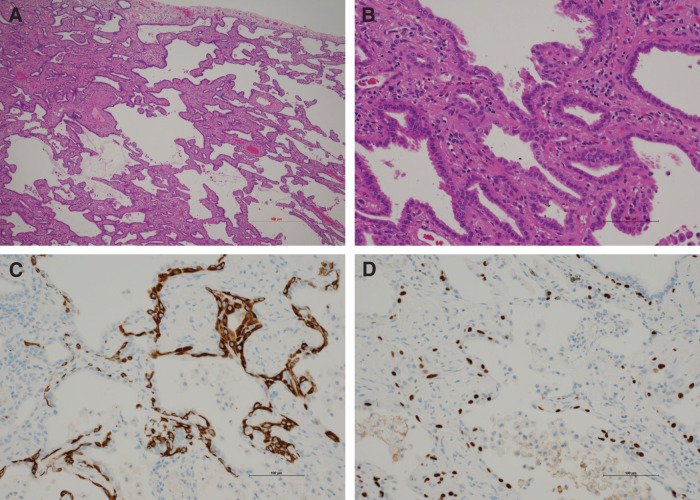
Histopathological and immunohistochemical findings in Case 2. (**A**) Low-power H&E staining (original magnification, ×40) showing a subtle bronchiolar-type epithelial proliferation spreading along the alveolar septa. (**B**) High-power H&E staining (×200) revealing bland cytology without appreciable atypia and focally interspersed ciliated cells. (**C**) CK5/6 immunostaining (×200) delineating the basal cell component as a continuous layer. (**D**) p40 immunostaining (×200) further confirming a continuous basal cell layer beneath the luminal epithelium. H&E, hematoxylin and eosin

A 77-year-old man presented with a 9-mm pure GGN in segment 8/9 (S8/9) of the right lower lobe (**Fig. 4**), which showed slight growth over a 2-year period. Serum tumor markers (CEA, CYFRA, and cancer antigen 19-9 [CA19-9]) were within the normal ranges. Therefore, early lung adenocarcinoma (cTisN0M0, cStage 0) was suspected. Following preoperative CT-guided marking, video-assisted thoracoscopic wedge resection of the right lower lobe was performed. On the permanent sections, there was no significant cytological atypia, and ciliated epithelium was present only focally (**Fig. 5A** and **5B**). Immunohistochemical analysis using CK5/6 and p40 confirmed the presence of a continuous basal cell layer (**Fig. 5C** and **5D**), and BA/CMPT was diagnosed. The postoperative course was uneventful, and the patient was discharged on POD 10. He is currently being followed up without treatment and has remained recurrence-free for 3 months after the surgery.

## DISCUSSION

BA/CMPT originated from the concept of the CMPT, which was first reported by Ishikawa^1)^ in 2002, and was subsequently refined through the accumulation of additional cases and conceptual reorganization. It was recently described as a distinct entity in the 5th edition of the WHO Classification of Thoracic Tumours (2021). The reported sex distribution is approximately equal. Most patients are aged 60–80 years, although cases involving adolescents have also been reported.^2)^ BA/CMPTs typically present as solitary peripheral lesions located subpleurally or near the pleura, with a predilection for the right lung and lower lobes.^2)^ The reported tumor size ranges from 2 to 45 mm, with most lesions measuring <20 mm.^2,3)^ The reported growth rate is approximately 0.49 mm/year (range, 0–2 mm/year). On PET-CT, uptake tends to be low (SUVmax 0.57–1.35),^3)^ although a case with marked uptake (SUVmax 13.0) has also been reported. Cases showing high FDG uptake may tend to have larger tumor size than other BA/CMPTs.^4)^ Therefore, PET-CT alone is unlikely to reliably distinguish BA/CMPT from lung adenocarcinoma. Preoperative diagnoses of BA/CMPT are rare,^5)^ and surgery is often performed for both diagnostic and therapeutic purposes because lung cancer cannot be ruled out. Although both lesions were relatively small compared with many previously reported BA/CMPTs, they were within the known size range. Their radiologic appearances were contrasting, with a small solid nodule in Case 1 and a pure GGN in Case 2, and both lesions showed interval growth. Because the lesions were subcentimeter, peripheral, and located immediately beneath the pleura, preoperative histologic diagnosis was not readily achievable, which may have contributed to the diagnostic difficulty. Under these circumstances, because malignancy could not be excluded, wedge resection was considered an appropriate initial procedure for both diagnostic and therapeutic purposes.

Histologically, BA/CMPTs are composed of a bronchiolar type bilayered epithelium with a continuous basal cell layer.^6)^ In routine practice, diagnosis is based on histological findings and immunohistochemistry. The demonstration of a continuous basal cell layer using basal cell markers (e.g., p40 and CK5/6) is the most important diagnostic feature.^6,7)^ The absence of significant cytological atypia and the presence of mixed mucinous and ciliated cells further support this diagnosis. The most critical differential diagnosis is adenocarcinoma, particularly invasive mucinous adenocarcinoma, which often exhibits only mild cytological atypia and may be difficult to distinguish from BA/CMPTs on morphological grounds alone.

During intraoperative frozen-section consultations, cilia can be difficult to identify because of freezing artifacts and suboptimal specimen conditions. In addition, assessment of the continuity of the basal cell layer is often limited.^7)^ In Case 1, ciliated cells were not detected on the frozen sections (**Fig. 2A**), leading to misdiagnosis as adenocarcinoma. Notably, ciliated cells became identifiable after formalin fixation of frozen tissue sections (**Fig. 2B**), highlighting the limitations of frozen-section evaluation. Previous studies have also emphasized that distinguishing BA/CMPTs from adenocarcinomas during intraoperative consultation is challenging, even for experienced thoracic pathologists, potentially leading to overtreatment.^8)^ This diagnostic pitfall is particularly problematic in lesions with abundant mucin, from which invasive mucinous adenocarcinomas cannot be confidently excluded. Some reports have suggested that diagnostic accuracy improves in institutions with prior experience of BA/CMPTs.^5)^ Therefore, broader recognition of this entity is warranted. Furthermore, it has been reported that BA/CMPT should be considered in the differential diagnosis when a peripheral pulmonary nodule is located in the basal segment of the lower lobe, which exhibits features such as an irregular shape, shows a distance from the lesion edge to the pleura of ≤5 mm, lacks a spiculation sign, or is accompanied by pseudo-cavitation.^9,10)^ Therefore, sharing these imaging findings, together with preoperative clinical information such as the patient’s background and clinical course, with pathologists may contribute to improving the diagnostic accuracy of BA/CMPT. In addition, we believe that, during intraoperative frozen section diagnosis, careful attention to the presence of a continuous basal cell layer may further aid in the diagnosis of BA/CMPT and help avoid overtreatment.

Surgical resection is the first-line treatment for BA/CMPTs. The prognosis is excellent, and recurrence or metastasis has rarely been reported. Because BA/CMPTs predominantly occur in the peripheral lung and are considered low-grade, wedge resection is generally sufficient, and extensive resection and systematic lymph node dissection are not required.^11)^ Most reported BA/CMPTs have been managed with limited resection, particularly wedge resection. However, lobectomy has also been performed in cases in which malignancy could not be excluded preoperatively or on intraoperative frozen section.^12)^ Regardless of whether lobectomy or limited resection was performed, the prognosis of BA/CMPT has been consistently favorable. Although no standardized follow-up duration has been established, complete resection appears to be associated with a favorable course, and a follow-up of up to 5 years has been considered sufficient in published reports.^2,11)^

BA/CMPTs can exhibit diverse radiological appearances ranging from a small solid nodule to a pure GGN. Differences in appearance have been attributed to the amount of mucin within the tumor; mucin-rich lesions may present as pure or mixed GGNs, whereas mucin-poor lesions may appear as solid nodules.^3)^ However, in our cases, the relationship was reversed: the solid nodule (Case 1) contained abundant mucin, whereas the pure GGN (Case 2) contained less mucin. These findings suggest that CT appearance may be influenced by multiple factors in addition to mucin content, such as aeration, cellularity, and stromal changes, warranting further investigation. Molecular studies have demonstrated that BA/CMPTs can harbor driver alterations, including EGFR, KRAS, and BRAF mutations, with BRAF alterations reported frequently.^2,6)^ Although molecular findings are not mandatory for diagnosis, they contribute to a better understanding of the disease and may aid in the differential diagnosis.

## CONCLUSIONS

Here, we report two cases of resected BA/CMPTs with distinct radiological morphologies. Although BA/CMPTs are rare, they may be misdiagnosed as adenocarcinomas during intraoperative frozen-section consultation. Unrecognized cases may lead to overtreatment, such as lobectomy and systematic lymph node dissection. Shared awareness of this condition and preoperative communication of clinical information with pathologists are important to avoid overtreatment. Further accumulation of cases is required to clarify the diagnostic criteria and optimize management strategies for BA/CMPTs.

## DECLARATIONS

### Availability of data and materials

Data sharing is not applicable to this article as no datasets were generated or analyzed.
